# Comparison of Peak Cardiopulmonary Performance Parameters on a Robotics-Assisted Tilt Table, a Cycle and a Treadmill

**DOI:** 10.1371/journal.pone.0122767

**Published:** 2015-04-10

**Authors:** Jittima Saengsuwan, Tobias Nef, Marco Laubacher, Kenneth J. Hunt

**Affiliations:** 1 Institute for Rehabilitation and Performance Technology, Division of Mechanical Engineering, Department of Engineering and Information Technology, Bern University of Applied Sciences, Burgdorf, Switzerland; 2 ARTORG Center for Biomedical Engineering Research, Gerontechnology and Rehabilitation Research Group, Bern University, Bern, Switzerland; 3 Department of Physical Medicine and Rehabilitation, Faculty of Medicine, Khon Kaen University, Khon Kaen, Thailand; Fondazione G. Monasterio, ITALY

## Abstract

Robotics-assisted tilt table (RATT) technology provides body support, cyclical stepping movement and physiological loading. This technology can potentially be used to facilitate the estimation of peak cardiopulmonary performance parameters in patients who have neurological or other problems that may preclude testing on a treadmill or cycle ergometer. The aim of the study was to compare the magnitude of peak cardiopulmonary performance parameters including peak oxygen uptake (VO_2peak_) and peak heart rate (HR_peak_) obtained from a robotics-assisted tilt table (RATT), a cycle ergometer and a treadmill. The strength of correlations between the three devices, test-retest reliability and repeatability were also assessed. Eighteen healthy subjects performed six maximal exercise tests, with two tests on each of the three exercise modalities. Data from the second tests were used for the comparative and correlation analyses. For nine subjects, test-retest reliability and repeatability of VO_2peak_ and HR_peak_ were assessed. Absolute VO_2peak_ from the RATT, the cycle ergometer and the treadmill was (mean (SD)) 2.2 (0.56), 2.8 (0.80) and 3.2 (0.87) L/min, respectively (p < 0.001). HR_peak_ from the RATT, the cycle ergometer and the treadmill was 168 (9.5), 179 (7.9) and 184 (6.9) beats/min, respectively (p < 0.001). VO_2peak_ and HR_peak_ from the RATT vs the cycle ergometer and the RATT vs the treadmill showed strong correlations. Test-retest reliability and repeatability were high for VO_2peak_ and HR_peak_ for all devices. The results demonstrate that the RATT is a valid and reliable device for exercise testing. There is potential for the RATT to be used in severely impaired subjects who cannot use the standard modalities.

## Introduction

Maximal oxygen uptake (VO_2max_) or peak oxygen uptake (VO_2peak_) is commonly used for the evaluation of physical fitness and for exercise prescription [1–3]. The most commonly used devices are treadmills and cycle ergometers. The VO_2max_ achieved from cycle ergometry has been observed to be 6–23% lower than from a treadmill [4–6].

There are some limitations to the use of standard devices in neurological patients who have weakness or coordination problem caused by stroke, multiple sclerosis or spinal cord injury [1]. The alternative recommended devices for these patients are a semi-recumbent cycle ergometer or a total body stepper [1], but severely affected patients have limitations that preclude them from using even these devices.

Recent systematic reviews have highlighted the importance of maintaining cardiorespiratory fitness after stroke [7] and spinal cord injury [8], but also emphasise the technical difficulty of implementing testing protocols and training programmes in these populations. Smith et al. included 42 studies in their systematic review of cardiorespiratory fitness after stroke and reported that VO_2peak_ was as low as 26% of that of healthy age- and gender-matched individuals; but, importantly, they noted that "most studies recruited patients with mild stroke" and pointed out that cardiorespiratory fitness is likely substantially lower in more severely affected patients [7]. The reason for inclusion of only mildly-affected patients in the reviewed studies is clear: most studies estimated VO_2peak_ using either a cycle ergometer or a treadmill, exercise modalities which are only usable in the case of mild to moderate impairment.

A robotics-assisted tilt table (RATT) provides the advantage of body support, cyclical stepping and physiological loading for early rehabilitation. These features are necessary for using the RATT for exercise testing and training in patients with severe disability: the body support makes it feasible and safe for patients with severe weakness or balance problems to exercise because the stability of the body is not required; thigh cuffs and foot plates stabilise the weak or spastic extremities and hold them in place. This type of device has been augmented by adding force sensors, work rate calculation and a visual feedback system to guide exercise intensity for exercise testing [9]. In a previous study, it was shown that it is feasible to measure peak cardiopulmonary performance parameters using the augmented RATT [10, 11]. To verify that the device can be used to measure peak cardiopulmonary performance parameters, the RATT should first be compared with the standard testing devices using able-bodied subjects.

The aim of this study was to compare the magnitude of peak cardiopulmonary performance parameters including peak oxygen uptake (VO_2peak_) and peak heart rate (HR_peak_) obtained from the RATT, a treadmill and a cycle ergometer. The strength of correlations between the devices, test-retest reliability and repeatability were also assessed.

## Materials and Methods

### Subjects and general study design

The study was reviewed and approved by the Ethics Review Board of the Canton of Bern in Switzerland (Reference No. 002/12). Written informed consent was obtained from all subjects prior to participation. The study was conducted in Bern University of Applied Sciences from December 2012 to September 2013.

Eighteen normal subjects (10 male, 8 female; Table 1) completed the study by performing 6 maximal exercise tests, with 2 tests on each of the three exercise modalities as described below. No subjects had cardiovascular, pulmonary or musculoskeletal problems that might have interfered with or contraindicated exercise testing.

**Table 1:** Baseline characteristics of subjects (n = 18).

Characteristic	Value—mean (SD)
Age [years]	28.6 (6.3)
Male/Female [n]	10/8
Smoking [%]	11.1
Height [cm]	172.4 (9.9)
Body mass [kg]	69.1 (12.8)
Body mass index [kg/m^2^]	22.7 (2.2)
Activity level [14]*	3.4 (1.1)

* level 1: inactive or little activity; level 2: regular (≥ 5 days/ week), low level of exertion (≥ 10 min at a time); level 3: aerobic exercise for 20–60 min/week; level 4: aerobic exercise for 1–3 hours/week; level 5: aerobic exercise over 3 hours/week.

Subjects performed a total of 6 tests using a treadmill (Venus, h/p/cosmos GmbH, Germany—2 tests), a cycle ergometer (LC7, Monark Exercise AB, Sweden—2 tests) and a RATT (Erigo, Hocoma AG, Switzerland—2 tests). The order of presentation of the tests for each subject was done by computer randomization and the subjects did not know in advance which testing device would be used. Each individual test was done on a separate day, and each test session was separated by at least 48 hours but not more than 7 days. The time of day for testing was the same for each subject. Participants were instructed to avoid strenuous activity within the 24 hours before testing and not to consume food, nicotine or caffeine at least three hours prior to testing [12, 13].

### Experimental procedures

Each incremental exercise test had the same structure. There was 3 minutes of rest, 5 minutes of warm up, a further 3 minutes of rest, and 3 minutes of unloaded movement. The ramp phase followed. Individualized, predicted maximum work rates for the treadmill and the cycle ergometer were calculated based on estimation of VO_2max_ [14] and the VO_2_-WR relationship [4]. For the RATT, individual maximum work rates were estimated using pilot data for healthy subjects [10]. The rate of increase in work rate was then calculated for each subject to achieve the predicted peak work rate in 10 minutes. Subjects then exercised until they reached their maximal performance and could not maintain the target work rate. Subjects were verbally encouraged to exercise to their limit of functional capacity.

#### RATT

**Fig 1:**
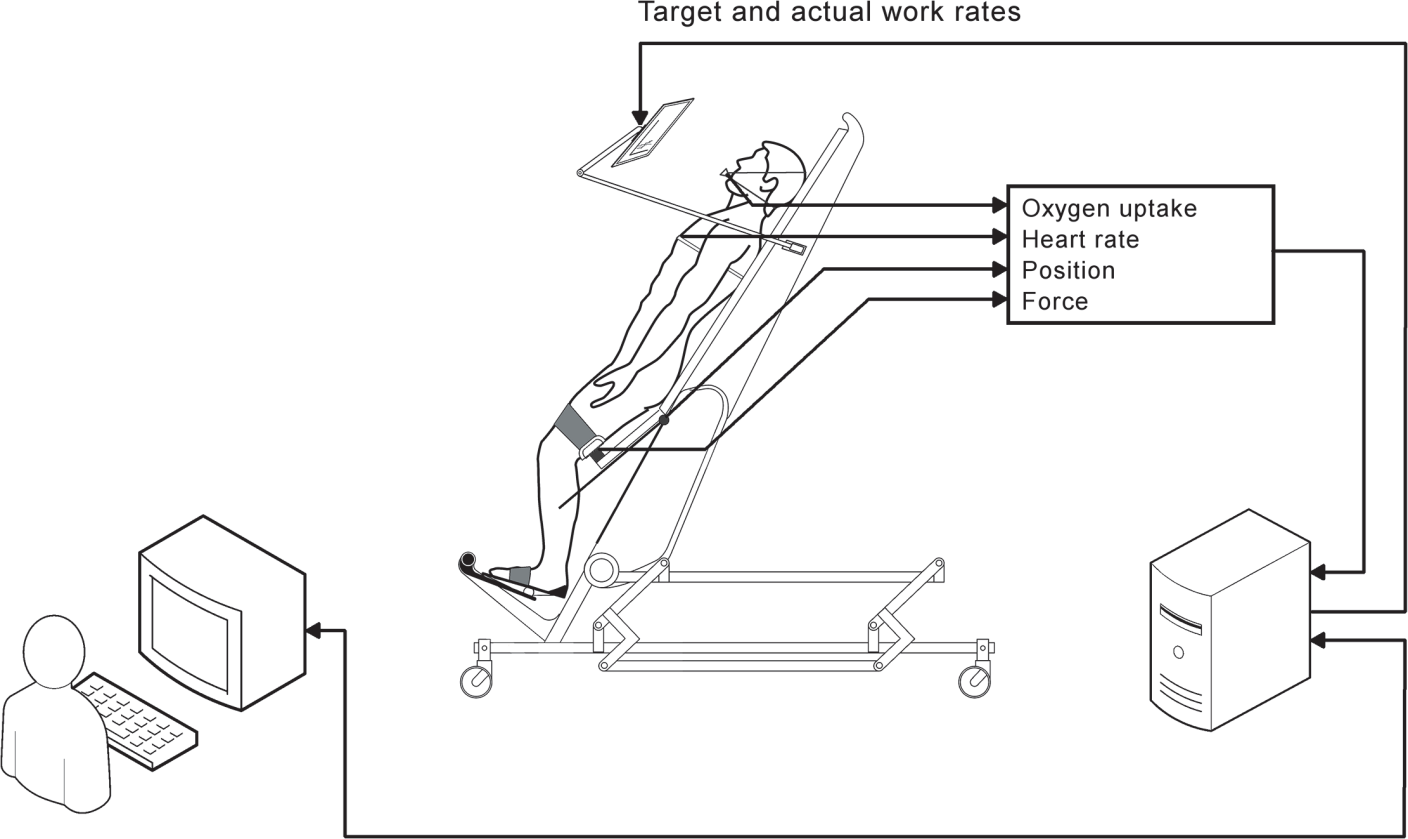
Work rate estimation and visual feedback. The subject's work rate is estimated continuously from forces in the thigh cuffs and joint angular velocities. A target work rate profile is displayed with the estimated work rate and the subject must adapt volitional muscular work to maintain the target. Physiological variables are monitored continuously.

The subjects were first placed in a horizontal position on the tilt table and secured in accordance with the provisions of the support system. The thighs and the feet were fixed to the thigh cuffs and foot straps. The tilt table then was tilted to 70 degrees and the stepping movement was set at 80 steps/minute. Warm up involved active participation of the subject at a constant work rate of 15 W. Unloaded movement was achieved by subjects remaining passive while the RATT moved their legs, which was associated with a work rate of 0 W. The RATT ramp rate was individually set in the range 4 to 12 W/min to meet the target ramp duration. During the ramp phase, subjects were instructed to actively produce force by pushing into the leg cuffs. The target work rate and measured work rate were visually fed back to the subjects in real time on a computer screen (Fig 1).

#### Cycle ergometer

The ramp phase was implemented by linearly increasing the work rate on the electromechanical brake. The warm up phase was set at a constant work rate of 50 W. Unloaded movement was achieved by subjects cycling at 0 W. The cycle ramp rate ranged from 12 to 40 W/min. The cycle cadence throughout the test was freely selected but always above 60 rpm. The settings for the seat height, handlebar height and the seat to handlebar distance were adjusted to accommodate each subject. Each individual set up was recorded to ensure the same position in subsequent tests.

#### Treadmill

Unloaded work was implemented using a low treadmill speed (0.9 km/h) and zero slope. The warm up phase was set at a speed of 5 km/h and zero slope. During the ramp phase, work rate was increased linearly every 30 seconds using combined non-linear changes in speed and slope [15].

### Measurements

Cardiopulmonary response variables were monitored using a breath-by-breath system (MetaMax 3B, Cortex Biophysik GmbH, Germany). The device was calibrated prior to each test for volume and gas concentration using a 3-L syringe and a precision gas calibration mixture (15% O_2_ and 5% CO_2_). Heart rate was continuously measured using a chest belt (T31, Polar Electro, Finland) and recorded directly on the MetaMax system. Additionally, on the RATT, the heart rate was recorded using a receiver board (HRMI, Sparkfun, Boulder, USA). Subjects rated perceived exertion and leg fatigue every 3 minutes during the incremental exercise test using the Borg CR10 scale for dyspnea and leg fatigue [16, 17].

### Outcome measures

Cardiopulmonary performance parameters were evaluated as follows: VO_2peak_ was obtained from a 30-second moving average of VO_2_. Peak respiratory exchange ratio (RER_peak_) was the average value of RER during the same period. Peak heart rate (HR_peak_) was the maximal heart rate value reached during the incremental phase. VE_peak_ was a 30-second average of the peak minute ventilation. Peak work rate (WR_peak_) was calculated from a 10-second average of the recorded work rate. The peak Borg CR10 scale for both dyspnea and leg fatigue were those recorded at the time that subjects reached their maximal performance. Time to VO_2peak_ and the reasons for test termination were also recorded.

### Statistical analysis

Data from the second test from each device were used for the comparative analysis among the three modalities. Normality of the data was assessed by the Kolmogorov-Smirnov test. Repeated measures analysis of variance (ANOVA) was conducted to determine whether there were significant differences between the peak cardiopulmonary performance parameters. If Mauchly’s test of sphericity was significant (p<0.05), Greenhouse-Geiser corrections were used. Bonferroni post-hoc multiple comparison corrections were applied to examine the differences between each paired data set, if a significant F ratio was found.

For correlation analysis, linear regression of the VO_2peak_ and HR_peak_ values for the RATT vs cycle ergometer and the RATT vs treadmill was performed. The regression equation, the correlation coefficient (R), the coefficient of determination (R^2^) and the standard error of estimate (SEE) were computed.

Test-retest reliability of VO_2peak_ and HR_peak_ on each device was analyzed using a 2-way, random intraclass correlation coefficient (ICC_2,1_) and a 95% confidence interval (CI). The within-subject variation of VO_2peak_ and HR_peak_ was calculated using the coefficient of variation [18]. The Bland and Altman limits of agreement were used to investigate the repeatability of VO_2peak_ and the HR_peak_ on each device. All analyses were performed using SPSS (Version 19.0, IBM Corp.).

During the first series of tests with each device, technical problems with the VO_2_ measurement device were detected in 9 subjects. Thus the comparative analysis was carried out using only data from the second series (all 18 subjects), and test-retest analysis was based on only 9 subjects.

## Results

### Comparison of peak values

Overall, statistically significant differences in all peak performance parameters, except in the Borg CR10 scale for leg effort, were seen between the RATT, the cycle ergometer and the treadmill (Table 2).

**Table 2:** Peak performance values from the RATT, cycle and treadmill (n = 18).

**Variables**	**RATT**	**Cycle ergometer**	**Treadmill**	**P value**
VO_2peak_ absolute (L/min)^a^ ^,^ ^b^ ^,^ ^c^	2.24 ± 0.13	2.81 ± 0.19	3.19 ± 0.20	<0.001
VO_2peak_ relative (mL/kg/min) ^a^ ^,^ ^b^ ^,^ ^c^	32.3 ± 4.9	40.2 ± 7.0	45.9 ± 7.6	<0.001
HR_peak_ (beats/min) ^a^ ^,^ ^b^ ^,^ ^c^	168.0 ± 9.5	178.8 ± 7.9	183.8 ± 6.9	<0.001
Percent predicted HR_peak_ (%)^a^ ^,^ ^b^ ^,^ ^c^	87.8 ± 5.3	93.5 ± 4.8	96.1 ± 4.2	<0.001
RER_peak_ ^a^ ^,^ ^b^	1.03 ± 0.1	1.13 ± 0.1	1.11 ± 0.1	<0.001
VE_peak_ (L/min) ^a^ ^,^ ^b^ ^,^ ^d^ (n = 17)	72.2 ± 21.1	101.4 ± 31.0	106.1 ± 32.0	<0.001
Borg CR10 scale dyspnea ^b^ ^,^ ^d^	6.6 ± 2.0	7.6 ± 1.7	9.1 ± 0.6	<0.001
Borg CR10 scale leg effort	8.8 ± 1.4	9.0 ± 1.6	9.1 ± 1.0	0. 65
WR_peak_ (W) ^a^ ^,^ ^b^ ^,^ ^c^	65.9 ± 18.0	233.5 ± 72.7	205.9 ± 70.1	<0.001
Time to VO_2peak_ (min)	9.9 ± 1.0	9.7 ± 1.2	9.0 ± 1.1	0.063

Data are given as mean ± standard deviation. VO_2_ = oxygen uptake, VO_2peak_ = peak oxygen uptake, HR_peak_ = peak heart rate, Percent predicted HR_peak_ = the peak heart rate expressed as a percentage of the predicted peak heart rate, RER_peak_ = peak respiratory exchange ratio, VE_peak_ = peak minute ventilation, WR_peak_ = peak work rate.

^a^ p < 0.001 between the RATT and the cycle ergometer

^b^ p < 0.001 between the RATT and the treadmill

^c^ p < 0.001 between the cycle ergometer and the treadmill

^d^ p< 0.05 between the cycle ergometer and the treadmill.

Absolute VO_2peak_ from the RATT, the cycle ergometer and the treadmill was (mean (SD)) 2.2 (0.56), 2.8 (0.80) and 3.2 (0.87) L/min, respectively (p < 0.001). Absolute VO_2peak_ obtained from the RATT was on average 19.0% lower than the cycle ergometer and 29.2% lower than on the treadmill.

HR_peak_ from the RATT, the cycle ergometer and the treadmill was 168 (9.5), 179 (7.9) and 184 (6.9) beats/min, respectively (p < 0.001). HR_peak_ obtained on the RATT was on average 6.0% lower than the cycle ergometer and 8.6% lower than on the treadmill.

The three most common reasons given by the subjects for stopping the RATT test were leg fatigue (66.7%), generalized fatigue (11.1%) and leg discomfort at high work rate (11.1%). Two subjects reported foot pain due to tight foot strap fixation, which immediately resolved after the straps were released following the test. The main reasons for stopping the test on the treadmill were breathing effort (44.4%), generalized fatigue (33.3%), and leg fatigue (16.6%). The main reasons for stopping the cycle test were leg fatigue (66.7%), generalized fatigue (16.7%) and breathing effort (11.1%). No other complaints or immediate complications after the exercise testing were observed.

### Correlation analysis

**Fig 2:**
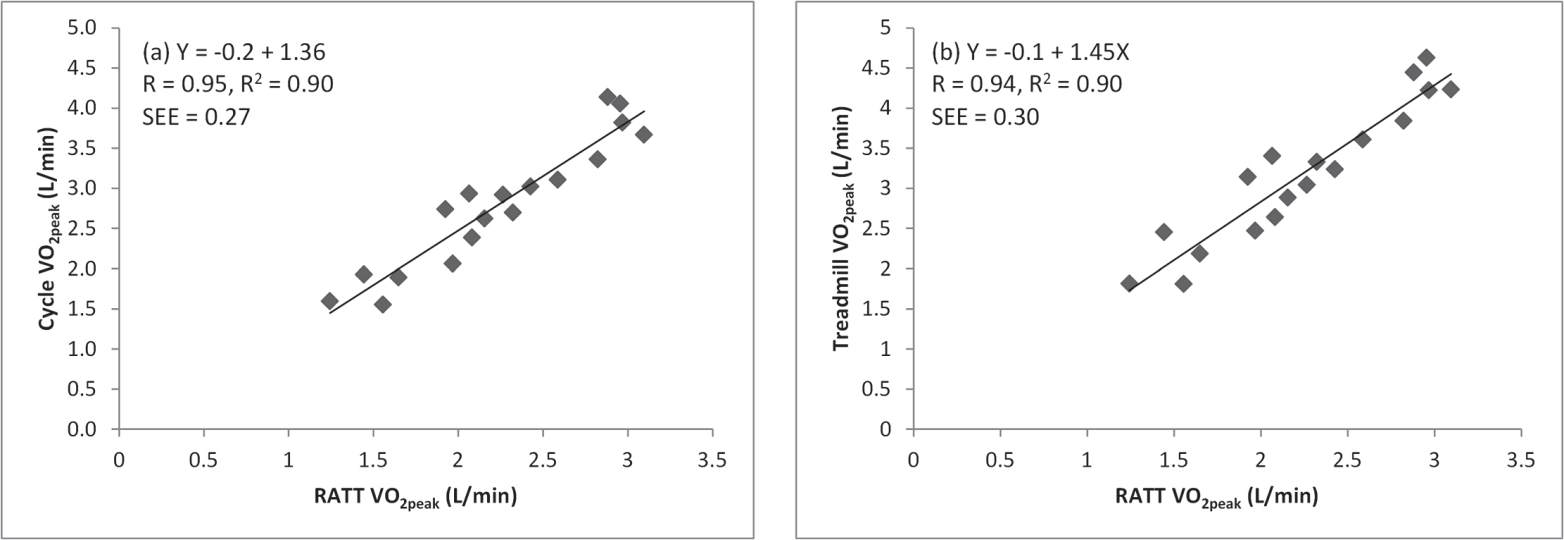
Linear regression analysis of VO_2peak_ (peak oxygen uptake): (a) RATT vs cycle, and (b) RATT vs treadmill. The equation, the correlation coefficient (R), the coefficient of determination (R^2^) and the standard error of estimation (SEE) are shown. The regression line is shown in each graph.

**Fig 3:**
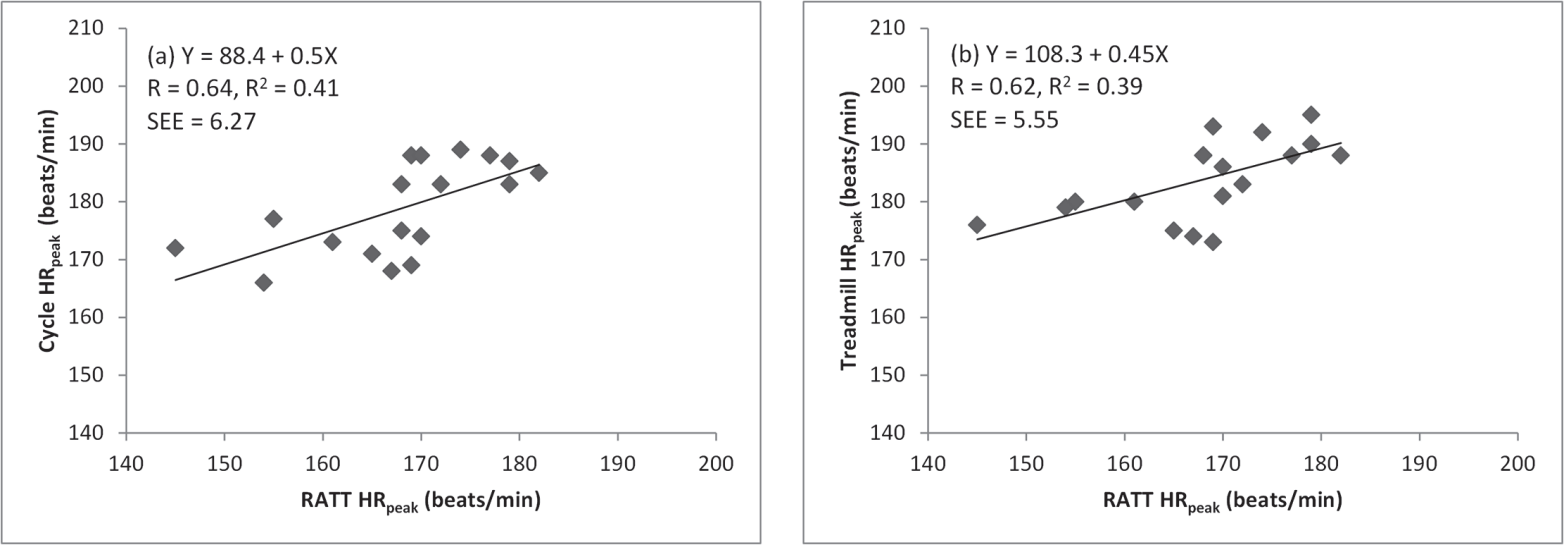
Linear regression analysis of HR_peak_: (a) RATT vs cycle, and (b) RATT vs treadmill. The equation, the correlation coefficient (R), the coefficient of determination (R^2^) and the standard error of estimation (SEE) are shown. The regression line is shown in each graph.

Linear regression analysis revealed very strong positive correlations between the RATT vs the cycle ergometer VO_2peak_ (r = 0.95. p<0.001) and the RATT vs the treadmill VO_2peak_ (r = 0.94. p<0.001) (Fig 2). There were strong positive correlation between the RATT HR_peak_ vs the cycle ergometer HR_peak_ (r = 0.64, p<0.005) and the RATT HR_peak_ vs the treadmill HR_peak_ (r = 0.62, p<0.05) (Fig 3).

### Test-retest reliability and repeatability

VO_2peak_ and HR_peak_ measured with all 3 devices had very high test-retest reliability with ICC_2,1_ ≥ 0.85 (Table 3). The coefficient of variation of the VO_2peak_ and HR_peak_ was less than 5% in all devices. The Bland and Altman analysis showed similar limits of agreement among the devices (Table 3).

**Table 3:** Test-retest reliability and repeatability of each device (n = 9).

	**Overall mean (tests 1 and 2)**	**MD (95% LoA)**	**CoV (%)**	**ICC (95% CI)**
VO_2peak_ (L/min)				
RATT	2.152	0.026 (-0.268, 0.320)	4.1	0.97 (0.89–0.99)
cycle ergometer	2.622	0.056 (-0.238, 0.342)	3.3	0.98 (0.94–1.00)
treadmill	2.924	0.013 (-0.271, 0.305)	2.4	0.99 (0.95–1.00)
HR_peak_ (beats/min)				
RATT	169.0	0.67 (12.57, -11.23)	1.8	0.89 (0.58–0.97)
cycle ergometer	180.3	2.56 (-5.77, 10.89)	1.6	0.86 (0.48–0.97)
treadmill	185.3	2.38 (-2.67, 7.33)	0.9	0.89 (0.40–0.98)

MD, mean difference; LoA, limits of agreement; CoV, coefficient of variation; ICC, intraclass correlation coefficient; CI, confidence interval; VO_2peak_, peak oxygen uptake; HR_peak_, peak heart rate.

## Discussion

The aim in the present study was to compare the magnitude of peak cardiopulmonary performance parameters including peak oxygen uptake (VO_2peak_) and peak heart rate (HR_peak_) obtained from the RATT, a treadmill and a cycle ergometer. It was also an aim to assess the strength of correlations between the devices, test-retest reliability and repeatability.

The results demonstrate that VO_2peak_ on the treadmill and the cycle ergometer is higher than on the RATT. On average, the VO_2peak_ values obtained from the RATT were 81.0% of the cycle ergometer VO_2peak_ and 70.8% of the treadmill VO_2peak_.

There were strong correlations between the RATT vs the cycle ergometer and the RATT vs the treadmill VO_2peak_. These results are comparable to the correlation of treadmill vs total body recumbent stepper VO_2peak_ (r = 0.92) [19] and the correlation of arm ergometer vs treadmill VO_2peak_ (r = 0.85) [20]. Both the cycle ergometer and treadmill have been validated as standard devices for estimation of peak cardiopulmonary performance parameters. The high correlation coefficients of VO_2peak_ between the devices investigated here suggests that the RATT, similarly, is a valid device for peak exercise testing within and between subjects. There is potential for the RATT to serve as an alternative to the cycle ergometer and treadmill for the estimation of VO_2peak_ in severely impaired subjects who cannot use the standard modalities.

An alternative device for investigation of cardiopulmonary performance in impaired subjects is the supine cycle ergometer [21]. Comparing the VO_2peak_ obtained from the RATT and the published data for the supine cycle ergometer, the RATT value is lower than the supine cycle ergometer: the supine cycle ergometer was approximately 22% lower than the treadmill VO_2peak_ in normal subjects [21]. The difference in the movement pattern on the RATT may account for the lower VO_2peak_ on the RATT. However, neurological patients who have severe weakness or spasticity may have difficulty pedaling on the supine cycle ergometer because there is no leg support.

The RATT appears to be able to provoke higher VO_2peak_ compared to arm ergometry. VO_2peak_ obtained from arm ergometry in healthy subjects was 42–43% lower than the treadmill VO_2peak_ [20] and 30–34% lower than the cycle ergometry VO_2peak_ [22–24]. Although the peak cardiopulmonary stress for the RATT is higher than for an arm ergometer, it is still lower than for a treadmill or cycle ergometer (VO_2peak_, HR_peak_ or RER_peak_). The lower cardiopulmonary stress may be explained by the lower level of muscle recruitment as a result of the body support and the differences in muscle mass used during the exercise, when compared to more physiological movement such as running or cycling [10].

Regarding test-retest reliability, the ICC for VO_2peak_ from each device is high. The lower limit of the 95% CI of the ICC for each device was more than 0.75, which is considered good reliability [25, 26]. Furthermore, the VO_2peak_ obtained from each device has high repeatability as determined by the Bland-Altman limits of agreement. The repeatability data were more precise than in a study of the repeatability of VO_2peak_ from the arm-leg ergometer as tested in healthy subjects (bias ± 1.96 SD = 0.016 ± 0.74 L/min) [27]. The within-subject coefficients of variation for VO_2peak_ and HR_peak_ were comparable to previous studies using cycle ergometry and treadmill exercise [28, 29].

HR_peak_ obtained from the RATT was lower than HR_peak_ from the treadmill and the cycle ergometer. Although strong correlations between the RATT vs cycle HR_peak_ and the RATT vs treadmill HR_peak_ were found, the correlation coefficient (R) and the coefficient of determination (R^2^) are lower compared to those for VO_2peak_. The R^2^ values found in this study (0.41 for the cycle, 0.39 for the treadmill) are slightly higher than in a study of Shrieks et al., who compared a treadmill with an arm crank ergometer and found that a linear regression for HR_peak_ for treadmill vs arm crank ergometer had R^2^ = 0.33 [20], which reflects that there are some factors which influence HR_peak_ other than the effect of the device itself. Previous work showed that age explained the majority of the variance [30, 31]. Other factors such as sex are controversial: Tanaka et al. stated that age predicted maximal heart rate to a large extent is independent of gender or physical activity status [30]; however, Faff et al. found a significant sex-dependent difference in the regression formula obtained after exercise on the treadmill and the cycle ergometer in athletes [32].

Repeatability of HR_peak_ from the RATT is comparable to the cycle ergometer. It was more precise compared to the study of Simmerlink et al., in which HR_peak_ repeatability from an arm-leg ergometer was 2.83 ± 19.85 beats/min [27]. Although the point estimates of ICC of the HR_peak_ from all devices studied here were high and comparable, the 95% CI were wide. Overall, the HRpeak parameter was seen to be less reliable than VO_2peak_.

A limitation of the present study is that, since a direct comparison between the devices in moderately or severely disabled neurological patients is not possible, it remains unknown whether the relative peak cardiopulmonary performance parameters can be extrapolated to the target patient population.

The data presented here, in particular the high correlation with standard devices and the high test-retest reliability and repeatability, support the validity and reliability of the RATT as a means of estimating peak cardiopulmonary performance parameters. The results demonstrate that the RATT has potential to be used for exercise testing in patients who have limitations for use of standard exercise testing modalities. The visual feedback system may be beneficial for the motivation of patients in both exercise testing and prescriptive exercise training. Future work should focus on the feasibility of peak cardiopulmonary performance testing using the RATT in populations with severe neurological impairments.

## Conclusions

The present study demonstrated that VO_2peak_ from the RATT was ∼20% lower than the cycle ergometer and ∼30% lower than the treadmill. The magnitude of difference is less than the arm ergometer [20, 23] but more than the supine cycle ergometer [21]. The high correlation coefficients, the high test-retest reliability and the high repeatability of the VO_2peak_ suggest that the RATT is a valid and reliable device for exercise testing. There is potential for the RATT to be used in severely impaired subjects who cannot use the standard modalities.
